# Examining *Felis catus* granulosa cell responses and extracellular vesicle characteristics under biomimetic gonadotropin stimulation in a microfluidic system

**DOI:** 10.3389/fcell.2026.1903837

**Published:** 2026-10-28

**Authors:** Kathryn M. Storey, Ahmed Gad, Mindy A. Meyers, Heloísa Coppini de Lima, Dawit Tesfaye, Nucharin Songsasen, Jennifer B. Nagashima

**Affiliations:** 1 Center for Species Survival, Smithsonian’s National Zoo and Conservation Biology Institute, Front Royal, VA, United States; 2 Animal Reproduction and Biotechnology Laboratory (ARBL), College of Veterinary Medicine and Biomedical Sciences, Colorado State University, Fort Collins, CO, United States; 3 Department of Animal Production, Faculty of Agriculture, Cairo University, Giza, Egypt; 4 Graduate Program in Wild Animals, School of Veterinary Medicine and Animal Science, São Paulo State University, São Paulo, CO, Brazil

**Keywords:** assisted reproductive technologies (ART), extracellular vesicles (EVs), gonadotropins, granulosa cell, microfluidics

## Abstract

**Introduction:**

Granulosa cells are essential regulators of follicular development and oocyte competence, yet the effects of dynamic gonadotropin exposure on granulosa cell function and extracellular vesicle (EV)-mediated communication remain poorly understood. This study aimed to develop a microfluidic platform that more closely mimics *in vivo* gonadotropin patterns of natural estrus and ovulation stimulation induction cycles, and investigated domestic cat granulosa cell responses under these controlled, dynamic conditions.

**Methods:**

Domestic cat granulosa cells were cultured in a microfluidic platform under dynamic gonadotropin profiles designed to replicate natural estrus and ovulation stimulation induction cycles. Cell viability and steroidogenic activity were assessed, and generated granulosa cell-derived EVs (gcEVs) were characterized by size, morphology, and tetraspanin (CD9 and CD81) expression relative to native follicular fluid EVs (ffEVs). Functional assays evaluated the effects of gcEVs produced under dynamic versus static gonadotropin treatments on gene expression in recipient granulosa cells.

**Result:**

Granulosa cells maintained in-chip viability greater than 88% and steroidogenic activity with the production of progesterone across all gonadotropin treatment groups. The gcEVs exhibited size, morphology, and CD9 and CD81 expression comparable to native ffEVs. Functional assays demonstrated that gcEVs generated under dynamic versus static gonadotropin regimens differentially modulated *CASP3* and *STAR* gene expression in recipient granulosa cells, whereas *CYP19A1* expression was unaffected.

**Discussion:**

These findings establish a granulosa cell-specific microfluidic model for investigating hormone-regulated EV communication under various gonadotropin exposure profiles. This platform provides a foundation for advancing our understanding of granulosa cell function and EV-mediated signaling, with potential applications in developing EV-based strategies to improve outcomes in assisted reproductive technologies.

## Introduction

Over 430,000 assisted reproductive technology (ART) cycles were reported in the United States in 2022 (CDC, 2024), representing a 126% increase in ART cycles over the last decade. While specific protocols vary across clinics, an ART cycle typically involves stimulation via exogenous gonadotropins - follicle stimulating hormone (FSH) to promote recruitment/development of additional ovarian follicles, followed by stimulation of final oocyte maturation via luteinizing hormone (LH) or human chorionic gonadotropin (hCG), with subsequent oocyte aspiration prior to ovulation. Oocytes are then cryopreserved or fertilized *in vitro* with resulting embryos cultured then either cryopreserved and/or transferred in a subsequent ART cycle. Yet despite broad use, our understanding of the impact of gonadotropins, and dynamic gonadotropin exposures in particular, on ovarian granulosa cells remains limited. This is crucial since bidirectional communication between granulosa cells and the oocyte underscores oocyte development, including regulating meiotic resumption, providing metabolic support, and protecting the oocyte from oxidative stress (Chauvin, 2025).

One type of communication between granulosa cells and the developing oocyte involves extracellular vesicles (EVs), or lipid membrane-bound vesicles secreted by all cells that include small EVs (<200 nm diameter) and large EVs (>200 nm diameter), though measured diameter is dependent on specific characterization methods (Welsh et al., 2024). EVs can originate through different cellular pathways, including budding from the plasma membrane or formation within the endosomal system (Doyle and Wang, 2019). EVs contain regulatory molecules, including proteins, peptides, RNA species, lipids, and DNA fragments (Bathala et al., 2018; Ferraz et al., 2019; Ranjbaran et al., 2019) which may be taken up by nearby cells via either fusion or endocytosis, contributing to cell-cell communication. EVs isolated from ovarian follicular fluid (ffEVs) have been demonstrated to stimulate granulosa cell proliferation (Hung et al., 2017) and cumulus cell expansion (Hung et al., 2015), modulate embryo epigenetic patterning (Silveira et al., 2017), and improve blastocyst production *in vitro* in the cow (Hung et al., 2015). In our laboratory, domestic cat ffEV supplementation during immature oocyte vitrification/rewarming enhances the oocyte’s ability to resume meiosis *in vitro* (de Almeida Monteiro Melo et al., 2020a). As such, ffEVs represent a compelling possibility for improving *in vitro* embryo production outcomes.

Clinical application of EVs for ARTs or other therapeutic needs requires use of clearly defined, consistently producible EVs at relevant concentrations (Ingato et al., 2016), ideally generated in controlled, *in vitro* conditions. *In vitro* production systems also reduce the risk of disease transfer and allow for the exploration of the mechanisms of EV generation. Toward these goals, bioreactor culture systems have been demonstrated to improve EV production rates compared with conventional static-incubation systems (Palviainen et al., 2019; Patel et al., 2018). Importantly, dynamic culture systems like bioreactors and organ-on-chip technologies allow for the control of flow and exposure of cells to growth factors, nutrients, and hormones. Unsurprisingly, EV production and cargo composition are under hormonal control (Almiñana et al., 2018; Burns et al., 2018; Greening et al., 2016; Fereshteh et al., 2018; de Ávila et al., 2020). In the cow, oocyte *in vitro* maturation in the presence of ffEVs from early luteal stage follicles versus late luteal resulted in upregulation of cumulus oocyte complex (COC) genes associated with gamete development, ovulation, and NOTCH signaling (de Ávila et al., 2020). There is also a marked difference in the ffEV miRNAome of women treated with hCG to stimulate ovulation during an IVF cycle compared with GnRH agonist (Machtinger et al., 2023). Thus, microfluidic systems also offer a valuable opportunity to generate EVs *in vitro* and to recapitulate both the normal, pulsatile pattern of gonadotropins of a natural estrus cycle as well as ART cycle ovulation induction protocols under controlled conditions to understand how these exposures influence intrafollicular communication.

Although human oocytes can be made available through donation, the developmental potential of these samples cannot be tested due to ethical and legal concerns (de Melo-Martin and Cholst, 2008; Green, 2001). This limitation can be overcome by using animal models, some of which have been utilized to fill the current knowledge gap, such as livestock and murine models. Despite other available animal models, there is additional insight and translational benefits in the domestic cat (*Felis catus*) model for human and endangered felid applications. The cat is an excellent biomedical research model for the study of physiology, aging, cancer, and genetic diseases (Haskins, 2009; O’Brien, 2004; Chambers et al., 2015; Narfström et al., 2011; He et al., 2005), and shares many traits with humans, including displaying the highest level of synteny with humans among non-primate species (O’Brien, 2004). Further, there are several similarities in gamete characteristics between the cat and human (Thuwanut et al., 2016; Fujihara et al., 2018; Comizzoli et al., 2003; Nestle et al., 2012; Lee et al., 2018), including preovulatory oocyte size (avg. 110 μm (Reynaud et al., 2009) vs. 105 μm (Picton et al., 2008) in humans), chromatin configuration of the germinal vesicle (diffuse with a single nucleolar-like body in both species (Comizzoli et al., 2011; Combelles et al., 2002)), and time to first meiotic maturation *in vitro* (24 h (Comizzoli et al., 2003; Combelles et al., 2002)). Moreover, cat ovarian tissues from a highly heterogeneous population, which provides relevant biological variability as observed in human and endangered felid populations, are readily available from routine ovariohysterectomies.

In this study, we aim to establish and validate a microfluidic culture system that mimics *in vivo* gonadotropin exposure. Using the domestic cat model, we sought to characterize granulosa cell responses and granulosa cell extracellular vesicles (gcEVs) generated under natural estrus and ovulation induction mimetic gonadotropin exposure in a microfluidic system, and evaluate the biological activity of generated gcEVs in recipient granulosa cells. We hypothesized that dynamic gonadotropin exposure would support granulosa cell steroidogenic activity and elicit cellular responses distinct from sustained, consistent gonadotropin exposure.

## Materials and methods

### Reagents

All reagents are from Sigma-Aldrich, St. Louis, MO, United States, unless otherwise specified.

### Collection of ovaries

Ovaries were obtained from domestic cats with no known health conditions undergoing routine ovariohysterectomy at local veterinary clinics. Only ovaries from queens in heat containing antral-stage follicles were utilized and no ovaries were obtained from queens recently postpartum that had not begun another estrous cycle (Chatdarong et al., 2005; Mehl et al., 2017). Immediately after excision, ovaries were placed in either L-15 medium containing 30 μg/mL penicillin G potassium and streptomycin sulfate salts, and 8.8 μg/mL ascorbic acid, or Dulbecco’s Phosphate Buffered Saline (PBS) with 100 U/ml penicillin and 100 ug/ml streptomycin sulfate on ice. Tissues were transported to the laboratory and processed within 6 h of excision. Ovaries were dissected from uterine tissue and washed once in cold PBS before follicle isolation. A subset of ovaries was utilized in a preliminary experiment to identify the target size of antral stage follicles for ffEV and granulosa cell isolation (see Supplementary File 1) for subsequent studies.

### Isolation of follicles, granulosa cells, and ffEVs

Based on preliminary ffEV results (Supplementary File 1), antral stage follicles (≥1.5 mm) from a total of 29 domestic cats (aged 6 months–7 years, pooled into 5 biological replicates of 5–7 cats each) were mechanically separated from the surrounding cortical tissue as previously described (Nagashima et al., 2021). Briefly, using a scalpel and 20-gauge needle or fine-tip tweezers under a stereomicroscope, sized follicles were cut away from the cortical tissue. Isolated follicles were then washed by gentle swirling 1x in cold PBS to remove remaining loosely attached cells prior to mechanical puncture in fresh cold PBS to release COCs and follicular fluid for ffEV isolation. Granulosa cells were mechanically scraped from the inner walls of the follicle using a 20-gauge needle after follicle opening and released into PBS with the follicular fluid. Fluid was spun 700x*g* for 7 min at 4 °C to pellet granulosa cells, which were subsequently processed for cell culture. The ffEV-containing supernatant was filtered through a 0.22 μm filter, then centrifuged twice at 100,000x*g* for 70 min at 4 °C (Sorvall MTX Micro-Ultracentrifuge, Thermo Fisher Scientific, Waltham, MA, United States) (Welsh et al., 2024; Ferraz et al., 2019; de Almeida Monteiro Melo et al., 2020a). The isolated ffEVs were re-suspended in cold PBS and stored at −80 °C until use.

### Granulosa cell initial culture

Pelleted primary granulosa cells were re-suspended in Endothelial Growth Medium 2 (EGM2, PromoCell, Heidelberg, Germany) with 5% Fetal Bovine Serum (FBS, Gibco of Thermo Fisher Scientific) and assessed for initial viability. Only replicates with >30% primary cell viability at collection were included in the study (Zheng et al., 2019). Granulosa cells were cultured/expanded in the same medium at 38 °C and 5% CO_2_, and cryopreserved at both passages 1 and 2 (P1 and P2) in EGM2 supplemented with 10% dimethyl sulfoxide (DMSO) and 10% FBS until use as previously described (Marks et al., 2025). Suspended cells (1 × 10^6^ cells/mL) were placed in 1.5 mL cryotubes (Fisher Scientific), and the cryotubes were placed in a Corning® CoolCell® container (Corning, NY, United States). Cells were slowly cooled to −80 °C overnight and plunged into liquid nitrogen the next day for long-term storage.

### Microfluidic chip fabrication

Molds for PDMS microfluidic device fabrication were designed using Sketchup (Trimble, Westminster, CO, United States) and were comprised of a 1.2 cm diameter, 0.30 cm high central chamber, three inlet channels (two at 0.86 × 0.10 cm merging into a center channel at 0.87 × 0.20 cm), and one outlet channel (0.30 × 0.30 cm) (de Almeida Monteiro Melo et al., 2020b) (Figure 1A, Supplementary File 2). Molds were printed using a Formlabs Form 3 B 3D Dental Printer (Formlabs, Somerville, MA, United States) using Model Resin V3 (FLDMBE03, Formlabs), washed in isopropanol, and cured using Form Cure V1 (Formlabs) for 60 min at 60 °C (de Almeida Monteiro Melo Ferraz et al., 2020). A PDMS mixture (Sylgard 184, Dow Inc., Midland, MI, United States) with a pre-polymer:curing agent ratio of 10:1 was poured on the 3D-printed molds and partially cured for 40 min at 62 °C in an oven. The PDMS layer was removed from the mold, sides were cropped using a scalpel blade, and a 1.5-mm hole was created in the central chamber with a biopsy punch (Integra® Miltex®, York, PA, United States). Using a 25-gauge needle, holes were created in the bottom three inlets and top outlet for insertion of 23-gauge 1″ sterile blunt needles (SAI Infusion Technologies, Lake Villa, IL, United States) for later gas-permeable silicone Tygon® tubing attachment (0.02 in. ID, 0.06 in. OD, Cole-Parmer, Vernon Hills, IL, United States). The PDMS layer with central chamber opening and blunt needles inserted was lightly coated on four corners in pre-polymer:curing agent mixture to ensure proper adhesion to 60 mm cell culture treated petri dish (Corning®). The PDMS layer was sealed on all edges to the 60 mm petri dish, and blunt needle attachment points were secured using pre-polymer:curing agent mixture, then completely cured at 62 °C overnight. Before use for culture, the devices were washed with 3 mL PBS supplemented with 1% penicillin and streptomycin (Gibco) at a 2 μL/min flow rate (de Almeida Monteiro Melo et al., 2020b), dried, then UV-sterilized.

**FIGURE 1:**
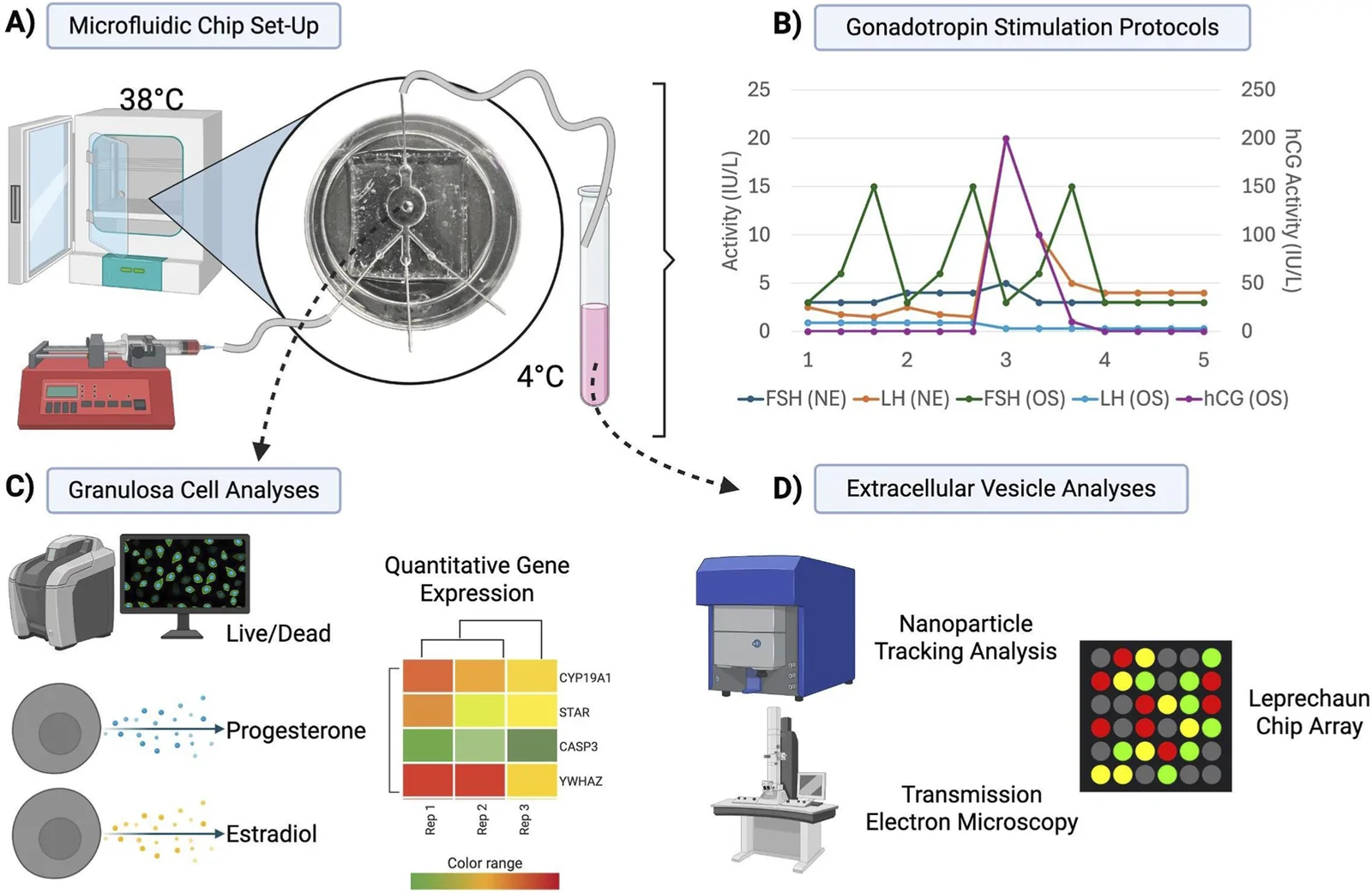
Overall study design, with **(A)** schematic of microfluidic chip set-up, demonstrating three lower chip channels connected via tubing to syringes containing medium and/or medium + hormones on syringe pump(s) (left and right most channels = hormone inputs, center channel = culture medium), center round cell chamber, and top output channel where spent medium flows through tubing connected to a collection tube in 4 °C refrigerator. All syringes pump at a cumulative rate of 2 µL per minute, and **(B)** graphs of each treatment group’s gonadotropin stimulation protocols (IU/L), **(C)** post-culture granulosa cell evaluations including live/dead counts, hormone concentration of progesterone and estradiol in spent media, and granulosa cell gene expression after co-incubation with gcEVs from the four different hormone treatment groups, and **(D)** EV evaluations including NTA, TEM and Leprechaun Chip Array. Created in BioRender. Storey, K (2026) https://BioRender.com/kfy7ogu.

### Biomimetic hormone stimulation in a microfluidic chip and gcEV generation

Granulosa cells were thawed, allowed to recover, then passaged (P2) at 3 × 10^4^ granulosa cells/chip into the center chamber of each microfluidic chip. Upon passage into chips, EGM2 supplemented with 5% EV-depleted FBS (-EV FBS; Gibco) was utilized for all treatment groups and herein referred to as culture medium. At 80% confluence, chips were then exposed to one of four treatment groups for 4.5 days (Figure 1B) based on *in vivo* human FSH and LH patterns and concentrations in both natural and ovarian-stimulation cycles. As intrafollicular FSH and LH concentrations across the feline reproductive cycle remain poorly characterized, human serum and natural cycle follicular fluid values were used to inform the gonadotropin exposure profiles in the current model. Initial concentrations were based on approximately half of reported serum values (von Wolff et al., 2014; Bomsel-Helmreich et al., 1979), with concentrations subsequently adjusted to reflect follicular fluid values measured at the time of oocyte retrieval, corresponding to day 4.5 in our culture system. Treatments included:

1. Control (C), no hormone supplementation
2. Baseline (B) hormone supplementation only (steady state), with 3 IU/L FSH and 2.5 IU/L LH
3. Natural estrus mimetic (NE) (Stricker et al., 2006), with FSH ∼3 IU/L at the onset of culture (equivalent to day −3 relative to the LH surge), then gradually elevating to 5 IU/L on day 3 of culture (∼day of the LH surge) before returning to original concentrations by day 4. LH levels of ∼2.5 IU/L at culture onset for a 12-h duration, before decreasing to 2 IU/L for another 12-h period, which continued for a subsequent 24 h. On day 3 of culture, LH sharply increased to 20 IU/L over 4 h as the LH surge, then decreased back to 4 IU/L until day 4.5 (equivalent to the time of ovum pickup).
4. Ovarian stimulation mimetic (OS) (Peñarrubia et al., 2003; Hu et al., 2020; Cabrera et al., 2002) started with 3 IU/L baseline FSH, introducing daily doses of 15 IU/L FSH (Smitz et al., 2007) (starting at 12 h and proceeding over 3 h, based on FSH half-life) (Muh and Oyesiku, 2012). LH was started at 0.9 IU/L (mimetic of conditions after typical GnRH-antagonist treatment) (Peñarrubia et al., 2003; Hu et al., 2020). On day 3 of culture, cells were exposed to 200 IU/L hCG (based on circulating serum hCG values within 8 h of a typical ovulation stimulating injection of 10,000 IU hCG) (Noorhasan et al., 2015).

Medium flow into the chip was controlled at 2 μL/min (Nagashima et al., 2018) via three autoclaved, gas-permeable silicone tubes into the inlet channels, attached to syringes (3 mL Monoject, Cardinal Health, Dublin, OH, United States and 20 mL Becton Dickinson, Franklin Lakes, NJ, United States) with 23-gauge 0.5″ blunt needles (SAI Infusion Technologies) containing culture medium, culture medium + recombinant human FSH (rFSH, R&D Systems, Minneapolis, MN, United States), or culture medium + purified human LH or hCG. Spent medium was collected in a 15 mL round-bottomed tube maintained at 4 °C, and processed at 48 h (pre-LH surge) and 108 h (end of incubation). Spent medium was pooled per treatment group, spun at 700x*g* for 7 min at 4 °C, and supernatant passed through 0.22 μm filter. Filtered supernatant was stored at −80 °C until EV isolation. At the time of use, supernatant was thawed overnight or for 24 h at 4 °C and spun 2x via ultracentrifugation at 100,000x*g* for 70 min at 4 °C. Pelleted gcEVs were re-suspended in cold PBS and further stored at −80 °C until use.

### Lipid droplet verification

To confirm lipid droplet identity of aggregated particulates observed in some granulosa cells following culture, Oil Red O staining was performed. Granulosa cells in static culture were incubated for 24 h in 400 µM oleic acid as a positive control (Yenuganti et al., 2016; Zhou et al., 2022). Briefly, a stock 0.5% w/v Oil Red O in isopropanol was diluted 3:2 in water and applied to paraformaldehyde-fixed granulosa cells from chips or positive control static culture for 10 min. Cells were washed with PBS and counterstained with hematoxylin for 1 min, washed with PBS until clear, then imaged at 20x under an EVOS Fl Auto 2 microscope (Thermo Fisher Scientific) (Yenuganti et al., 2016; BenchChem Technical Support Team, 2026).

### EV characterization via TEM, NTA, and Leprechaun Chip Assay

FfEVs and gcEVs (n = 3 and 5 biological replicates, respectively, 5–7 pooled cat samples per replicate) were evaluated via transmission electron microscopy (TEM, size/morphology) and nanoparticle tracking analysis (NTA, particle size/count). A subset of EVs (n = 1 each ffEV and gcEV) were further analyzed for biomarker expression using the Leprechaun Chip Assay from Unchained Labs (Pleasanton, CA, United States) with ExoViewR100. For TEM (Alpha Nano Tech, Durham, NC, United States), pooled ffEVs and gcEVs (by gonadotropin treatment group) (10 μL) were dropped onto Formvar-coated carbon-coated TEM grids (Electron Microscopy Sciences, Morgantown, PA, United States), allowed to be adsorbed to the grid for 10 min, and washed two times with water (30 s each). A 2% Aqueous Uranyl Acetate solution was applied to the grid for 30 s (negative staining of exosomes), then whisked off with filter paper, and grids were air-dried before imaging on a Jeol 1400 transmission electron microscope (Jeol United States Inc., Peabody, MA, United States).

NTA was performed using the ZetaView® QUATT 4 (Particle Metrix GmbH, Meerbusch, Germany), and data were analyzed via Software ZetaView® version 8.05.12 SP1 (Particle Metrix GmbH, Meerbusch, Germany) (Haines et al., 2025). Each ffEV sample (n = 3 biological replicates) and gcEV sample (n *=* 20 (4 treatments/replicate), N = 5 biological replicates) were diluted 1:10,000 or 1:100, respectively, in 0.22 μm filtered distilled water at room temperature and loaded into the cell, and the instrument measured each sample at 11 positions throughout the cell, via scatter mode using a 488 nm laser at each position. Acquisition parameters were set to a minimum brightness of 15, a maximum area of 1000 pixels, and a minimum area of 10 pixels (Helwa et al., 2017). Raw and summary data were exported to analyze particle diameter and concentration. The number of particles per particle size curves were created using GraphPad Prism (version 10.6.1 for Mac, GraphPad Software, Boston, Massachusetts, United States) (Helwa et al., 2017).

Tetraspanin CD9, CD63, and CD81 distribution was analyzed using the ExoViewR100 platform with the Leprechaun human tetraspanin ExoView kit (Unchained Labs Catalog number 251-1044) following the kit assay protocol. Briefly, EVs were isolated as described above, then processed through Exo-spin^TM^ size exclusion chromatography (SEC) columns to remove soluble proteins, contaminants, and smaller lipoproteins that may have aggregated with EVs during ultracentrifugation to enhance EV-only protein identification (Cell Guidance Systems, Babraham, Cambridge, United Kingdom). EVs were then diluted with the incubation solution and placed on top of the assay chips for the capture of EV-carrying mouse anti-human CD9 (Clone HI9a), mouse anti-human CD81 antigen (Clone JS-81), mouse anti-human CD63 (clone H5C6), and mouse isotype IgG1, k control (Clone MOPC-21). After 16 h incubation at room temperature chips were washed and then incubated with an antibody cocktail made of 0.6 μL mouse anti-human CD81 (Clone JS-81) conjugated with AlexFluor 555, 0.6 μL mouse anti-human CD63 (Clone H5C6) conjugated with AlexFluor 647, and 0.6 μL of mouse anti-human CD9 (Clone Hi9a) conjugated with AlexFluor 488 in 300 μL of blocking solution for 1 h at room temperature in on orbital shaker. Chips were washed and carefully dried. Image acquisition from each chip was carried out using the ExoView® R100 platform, and the data were analyzed by the ExoView Analyzer software version 3.2 (NanoView Biosciences, Boston, MA, United States). The images of the acquisition were visually inspected, and all artifacts on the spots were manually removed from the analysis. Non-specific binding was checked on the mouse isotype control IgG spots. The cutoff was manually established for all the chip to exclude the majority of the signal (>95%) captured on the isotype control.

### Automated immunoassays and liquid chromatography-tandem mass spectrometry

To confirm target microfluidic chip gonadotropin concentrations and assess granulosa cell responsiveness to stimulation, gonadotropin and steroid hormone concentrations in spent medium were assessed via automated immunoassay (AIA) and liquid chromatography-tandem mass spectrometry (LC-MS/MS) (Supplementary File 3), respectively (Bertoldo et al., 2019). FSH, LH, and hCG concentrations were measured by AIA on a Roche cobas e411 instrument (Roche Diagnostics, Indianapolis, IN, United States) using Roche assay reagents. Results were determined through an instrument-specific two-point calibration curve and a master curve provided through the reagent barcode. The assay ranges were as follows: FSH, 0.1–200 mIU/mL; LH, 0.1–200 mIU/mL; and hCG 0.1–10000 mIU/mL. Intra- and inter-assay coefficients of variation (CVs) for LH and FSH were determined by analysis of an in-house prepared human serum pool, and hCG CVs were determined using a Roche-supplied control based on human serum in two concentration ranges: LH (intra = 0.80%, inter = 4.76%); FSH (intra = 1.65%, inter = 3.86%); hCG QC1 (intra = 4.56%, inter = 4.16%); and hCG QC2 (intra = 0.78%, inter = 3.13%).

Progesterone (P4) and estradiol (E2) concentrations were quantified using LC-MS/MS. Analyses were conducted using a targeted assay based on previously published methods (Fecteau et al., 2024), employing reverse-phase chromatographic separation coupled to tandem mass spectrometric detection in multiple reaction monitoring (MRM) mode. Samples were injected at a volume of 25 μL, and calibration standards were prepared in solvent (25% methanol in water, v/v) rather than a biological matrix. Additional method parameters, including analyte-specific tuning and internal standard normalization, were performed according to the core facility’s validated protocols.

### Scratch test assay and cellular proliferation assessment

EV bioactivity was evaluated using a scratch assay to examine the impact on recipient granulosa cell viability and migration, and gene expression changes in recipient cells were also investigated. EVs were obtained from the aforementioned follicular fluid and chip-derived gcEVs. Fresh culture medium was processed identically to EV isolation via ultracentrifugation and served as a control (M_EV). P2 granulosa cells (n = 3 biological replicates) were seeded at a density of 2.5 × 10^3^ cells/well in 96-well plates (Thermo Fisher Scientific) for scratch assay and 1.25 × 10^3^ cells/mL for gene expression testing, then maintained in -EV FBS medium for 3 days to allow for recovery. Prior to treatment, all cells were washed once with pre-warmed PBS, and a scratch was made down the center of scratch assay wells using a P200 pipette tip. Scratch assay wells were washed again in pre-warmed PBS to remove displaced cells. All wells were then cultured in medium supplemented with 4 μg EV protein, as quantified using the Qubit fluorometric protein assay (Thermo Fisher Scientific) for 24 h (scratch assay) or 48 h (gene expression). Treatment groups included M_EV, ffEVs, and chip-derived gcEVs from the following conditions: control (C_gcEV), baseline (B_gcEV), natural estrus (NE_gcEV), and ovulation stimulation (OS_gcEV). Cell viability at 24 h was assessed using the ReadyProbes Cell Viability Imaging Kit (Blue/Green) (Thermo Fisher Scientific) on scratch assay wells according to the manufacturer’s instructions. Images were captured using EVOS at 20× magnification, and live versus dead cells were quantified using Celleste 6 Image Analysis Software (Thermo Fisher Scientific) by a blinded operator. Cell migration was assessed at 24 h using ImageJ 2 (version 1.53t, U.S. National Institutes of Health, Bethesda, MD, United States). For each well, a region of interest (ROI) was defined based on the scratch area at 0 h (t0). This ROI was then applied to the corresponding 24 h image for the same replicate and treatment. Cell migration was quantified by counting the number of cells within the ROI at 24 h. Wells for gene expression testing were washed with pre-warmed PBS and trypsinized (Gibco) at 48 h for collection, spun down at 700*xg* for 7 min, supernatant removed, and flash-frozen in liquid nitrogen, then stored at −80 °C until RNA isolation.

### RNA extraction and quantitative reverse transcription-polymerase chain reaction

Total RNA isolation was performed using RNeasy Plus Micro Kit with gDNA eliminator spin columns (Qiagen, Germantown, MD, United States), and concentration and purity were assessed via NanoDrop OneC Spectrophotometer (Thermo Fisher Scientific). Complementary DNA (cDNA) was synthesized (40 ng from each sample) using SuperScript IV First-Strand Synthesis System (Thermo Fisher Scientific) according to the manufacturer’s instructions and stored at −20 °C until quantitative polymerase chain reaction (qPCR) analysis. With the exception of caspase-3 (*CASP3*)*,* sequences of gene-specific primers were utilized from previously reported work in the domestic cat (Songsasen et al., 2017). *CASP3* primer sequences were designed using Primer-BLAST provided by the National Center for Biotechnology Information (NCBI) (Ye et al., 2012). All primer sequences used in the present study are shown in Supplementary Table S1. The relative expression of all genes was normalized to an endogenous control gene, tyrosine 3-monooxygenase/tryptophan 5-monooxygenase activation protein zeta (*YWHAZ*), which has previously been used for qPCR normalization in domestic cat ovarian cells and demonstrated stable expression in granulosa cells, plus greater expression stability relative to commonly used reference genes, including glyceraldehyde-3-phosphate dehydrogenase (*GAPDH*) (Songsasen et al., 2017; Lv et al., 2017). Each PCR (total volume 20 μL) consisted of 2 μL cDNA and 18 μL reaction mixture containing 10 μL FastStart Essential DNA green master (Roche), 2 μL of 10 μM forward and reverse combined primer mix, and 6 μL nuclease-free water. Reactions were performed with the following settings: 95 °C for 10 min, followed by 40 cycles at 95 °C for 15 s, specific annealing temperature (Supplementary Table S1) for 15 s, and 72 °C for 30 s. All amplifications were performed in duplicate using a LightCycler 96 (Roche). No template control reactions were performed in parallel as a negative control. Primer efficiency was assessed in each gene by serial dilution of DNA (Supplementary Table S1). Calculation of mRNA expression levels was performed by the comparative Ct method with *YWHAZ* as the reference gene. Granulosa cells exposed to M_EV were used as a control group for calculations of relative quantitation. Data are reported on a log_2_ scale as *n-*fold difference relative to the M_EV group. Calculations were performed using Microsoft Excel (Version 16.109) and Prism (version 10.6.1, GraphPad).

### Statistical analyses

All statistical analyses were performed using GraphPad Prism with three biological replicates per treatment group unless otherwise noted. Granulosa cell viability was analyzed using repeated-measures two-way ANOVA with Holm-Šídák correction. Total cell number per chip was analyzed using one-way ANOVA followed by Tukey’s multiple comparisons test. For NTA-derived parameters, statistical analyses were performed separately to assess differences among gcEV treatment groups and between gcEVs and ffEVs. Comparisons among matched gcEV treatment groups (n = 5 biological replicates/treatment group) were performed using a repeated-measures one-way ANOVA with the Geisser-Greenhouse correction for X10, X50, X90, span, mean particle size, and particle concentration, followed by Tukey’s multiple comparisons test. Because mode was not normally distributed, differences in mode among gcEV treatment groups were assessed using a Friedman test followed by Dunn’s multiple comparisons test. Comparisons between matched gcEVs and ffEVs were performed using a repeated-measures one-way ANOVA with Geisser-Greenhouse correction for X10, X50, X90, mean particle size, mode, and particle concentration, followed by Šídák’s multiple-comparisons test. Particle concentration data were log-transformed prior to analysis. Because span values were not normally distributed, differences in span values between gcEVs and ffEVs were assessed using a Friedman test followed by Dunn’s multiple comparisons test. Progesterone concentration in spent culture medium was analyzed using repeated-measures two-way ANOVA with Tukey’s *post hoc* test. Scratch migration data were analyzed using one-way ANOVA with Tukey’s *post hoc* test. Statistical comparisons between treatment groups for gene expression changes were performed using paired t-tests on ΔCt values. Normality was assessed using the Shapiro-Wilk test where applicable.

## Results

### Biomimetically stimulated granulosa cells are viable and respond to hormones

Granulosa cell viability was assessed in each gonadotropin treatment group (n = 3 biological replicates/treatment) after 4.5 days of exposure to gonadotropins and flow shear stress (Figure 2). Viability was highest in the B treatment with an average of 94.1% live cells (SD = ± 3.8), followed by NE (93.8% ± 2.3), OS (93.6% ± 7.8), and C (88.1% ± 7.4) (Figures 2A,B), though these were not statistically significant. No statistically significant differences were found in the total number of cells per chip at the end of incubation between treatment groups (Figure 2C), though control and ovulation stimulation treatment group chips displayed greater variations in cell number between replicates. Phase contrast imaging revealed possible lipid droplet accumulation post microfluidic culture in various hormone treatment groups (Figure 2A). Oil Red O staining was performed as a qualitative validation of intracellular lipid droplets observed following microfluidic granulosa cell culture. Lipid staining was confirmed in static granulosa cells, including oleic acid-BSA treated cells as a positive control, and in representative post-culture microfluidic samples from the C, B, and NE treatments (Supplementary File 4).

**FIGURE 2:**
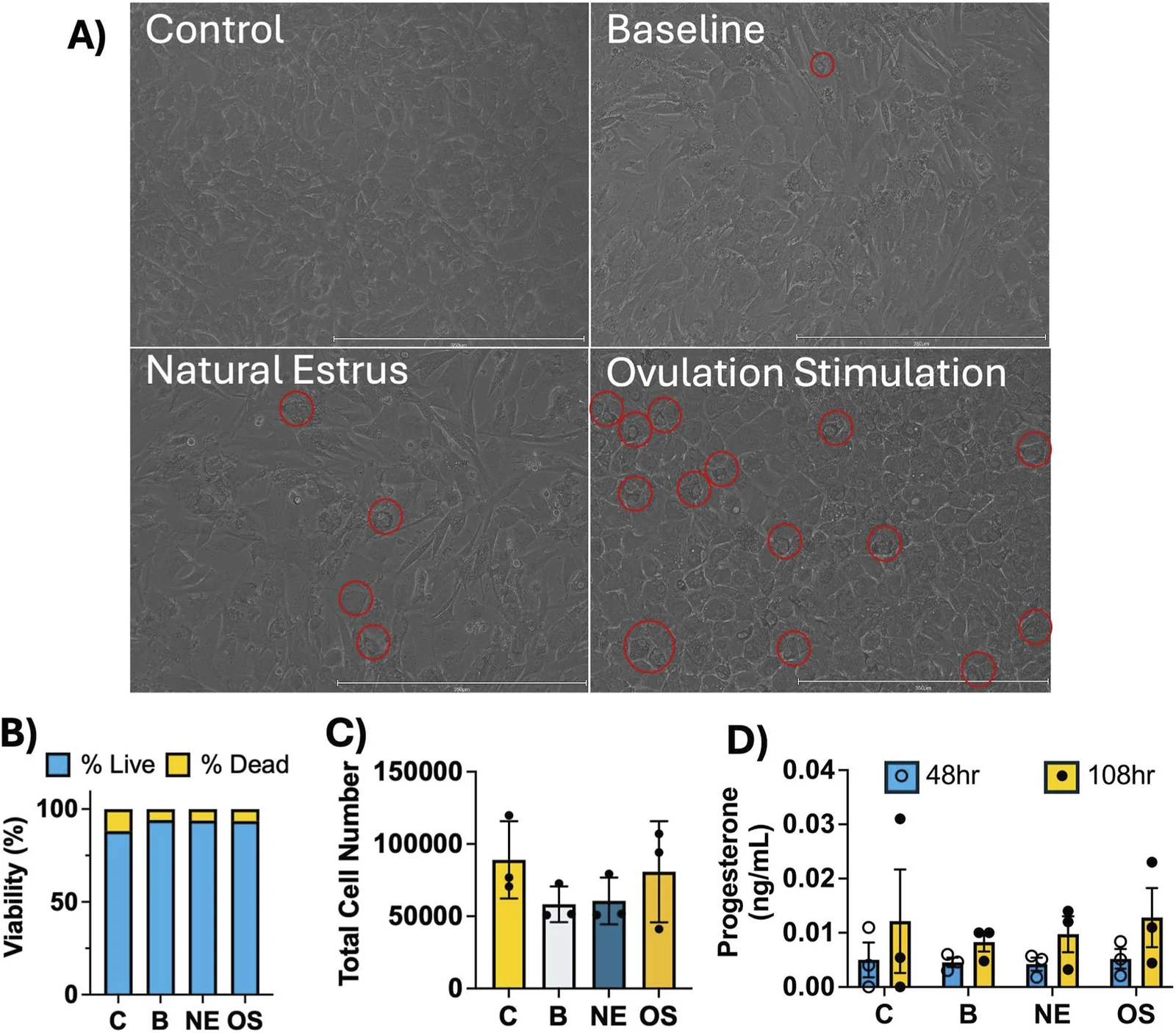
Characterization of domestic cat ovarian granulosa cells exposed to Control **(C)**, Baseline **(B)**, Natural Estrus-mimetic (NE), and Ovulation induction-mimetic (OS) gonadotropin protocols, with **(A)** representative phase contrast 20x images of post-incubation granulosa cell morphology in the microfluidic chamber for each gonadotropin treatment group, **(B)** post-incubation cell viability per treatment group (n = 3 biological replicates/treatment), **(C)** total cell number post-incubation (live and dead) across treatment groups (n = 3 biological replicates/treatment), and **(D)** progesterone concentration in spent medium after 48 and 108 h incubation (n = 3 biological replicates/treatment). Red circles denote observed lipid droplet accumulation. Total cell number graph is represented as mean ± SD and progesterone graph is mean ± SEM. Scale bars represent 350 µm.

Granulosa cell functionality was examined via progesterone (P4) and 17-β-estradiol (E2) concentration in spent culture medium during 48 vs. 108 h incubation (n = 3 biological replicates per treatment). A significant overall effect of time was observed for P4 production independent of treatment group, indicating that P4 production differed between 48 and 108 h across all treatment groups (*p* = 0.0233, Figure 2D), but no individual time-point comparisons reached significance following multiple-comparison correction. E2 was not detected for any time points or treatment groups (data not shown).

### Chip-derived gcEVs demonstrate ffEV-like characteristics

NTA analysis was used to characterize gcEVs across hormonal treatment conditions and compare particle characteristics among gcEV treatment groups relative to ffEVs. Particle characteristics included X10 (10% of particles in the distribution are below this size), X50 (median particle size), X90 (90% of particles in the distribution are below this size), span ((X90 – X10)/X50), mean and mode particle size, and particle concentration (particles/mL) (Table 1). There were no statistically significant differences in EV characteristics among treatment groups when comparing gcEV groups. When comparing matched gcEVs to native ffEVs, the span value significantly differed among groups (*p* = 0.0244), with baseline gcEVs exhibiting a significantly lower span, or narrower size range, compared with ffEVs (adjusted *p* = 0.0268). Additionally, ffEV concentration was significantly higher than all gcEV treatment groups (adjusted *p* = 0.0057 C vs. ffEV; 0.0014 B vs. ffEV; 0.0011 NE vs. ffEV; and 0.0006 OS vs. ffEV) with an average ffEV concentration of 1.4 × 10^11^ ± 9.3 × 10^10^ particles/mL (Figures 3A–E). FfEVs had a wider distribution of particle size compared to gcEVs, though mode sizes remained non-significantly different (Figures 3A–E; Table 1). Additionally, both -EV FBS and EGM2 were analyzed via NTA to determine background vesicle concentration in all treatment groups potentially derived from the commercial reagents. EGM2 particles were denoted as “Min Traces” on NTA, falling below the detectable limit. Thus, EV concentration was below the limit of detection or undetectable in the EGM2 sample. The -EV FBS sample contained particles on NTA, though they were smaller in size than generated gcEVs and native ffEVs (Table 1).

**TABLE 1:** GcEV and ffEV Nanoparticle Tracking Analysis results.

Treatment	x10 (nm)	x50 (nm)	x90 (nm)	Span	Mean (nm)	Mode (nm)	Concentration (particles/mL)
Control	62.6 ± 3.2	118.8 ± 5.5	207.7 ± 17.4	1.20 ± 0.07	144.3 ± 7.8	128.8 ± 3.3	4.6 × 10^9^ ± 2.1 × 10^9^ **
Baseline	61.3 ± 4.1	117.4 ± 5.1	200.2 ± 6.9	1.18 ± 0.08 *	141.9 ± 4.9	127.9 ± 4.6	4.8 × 10^9^ ± 2.2 × 10^9^ **
Natural estrus	59.9 ± 3.9	116.1 ± 7.3	198.7 ± 10.6	1.20 ± 0.1	140.0 ± 6.5	126.7 ± 6.8	3.8 × 10^9^ ± 1.5 × 10^9^ **
Ovulation stimulation	63.1 ± 4.7	116.1 ± 4.8	207.8 ± 8.5	1.28 ± 0.08	143.5 ± 5.4	126.4 ± 3.7	3.4 × 10^9^ ± 1.9 × 10^9^ ***
Follicular fluid EVs	77.4 ± 12.6	183.8 ± 40.0	377.7 ± 79.9	1.57 ± 0.06	226.7 ± 47.3	149.9 ± 126.3	1.4 × 10^11^ ± 9.3 × 10^10^
-EV FBS	40.7	83.9	151.9	1.30	106.5	97.8	1.5 × 10^10^

x10 = 10% of particles in the distribution are smaller than this size; x50 = median particle size in distribution; x90 = 90% of the particles are smaller than this size; and span = (x90 – x10/x50), which is a metric to demonstrate the breadth of particle distribution. Values represent the average across treatment groups (n = 5 biological replicates gcEVs; n = 3 biological replicates ffEVs) ± standard deviation. -EV FBS is a sample of the EV depleted fetal bovine serum used in the culture medium. * = p < 0.05; ** = p < 0.01; *** = p < 0.001 compared to follicular fluid EVs

**FIGURE 3:**
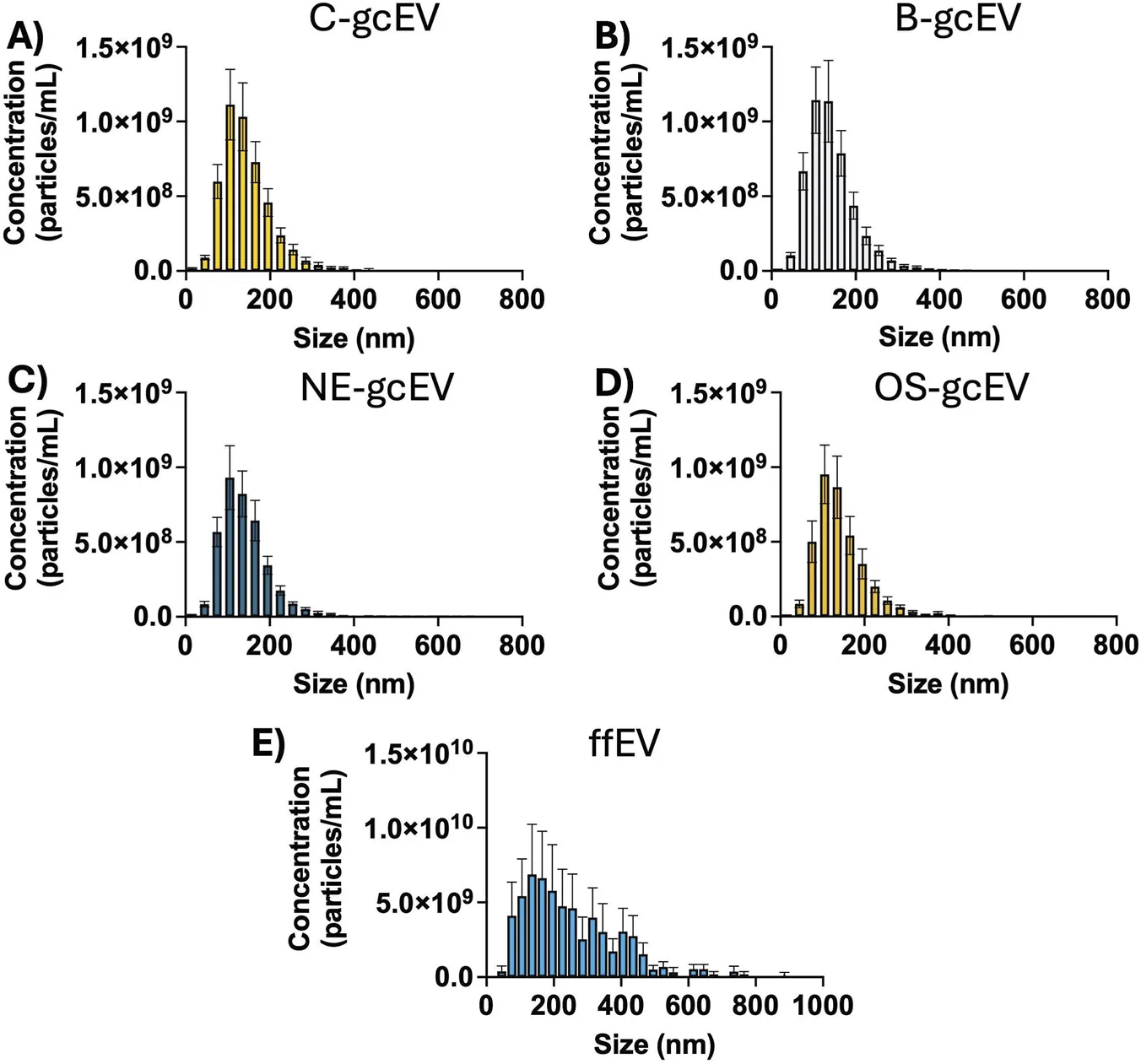
Nanoparticle Tracking Analysis characterization of domestic cat gcEVs derived from ovarian granulosa cells exposed to Control (C-gcEV), Baseline (B-gcEV), Natural Estrus-mimetic (NE-gcEV), and Ovulation induction-mimetic (OS-gcEV) gonadotropin protocols and corresponding ffEVs, with **(A–E)** NTA per treatment group (n = 5 biological replicates/treatment gcEVs and n = 3 biological replicates ffEVs). NTA graphs are represented as the mean concentration per size ±SEM.

TEM confirmed the characteristic presence of circular, doughnut-shaped vesicles in a heterogeneous, though narrow, size distribution for both ffEVs and gcEVs (Figures 4A,B).

**FIGURE 4:**
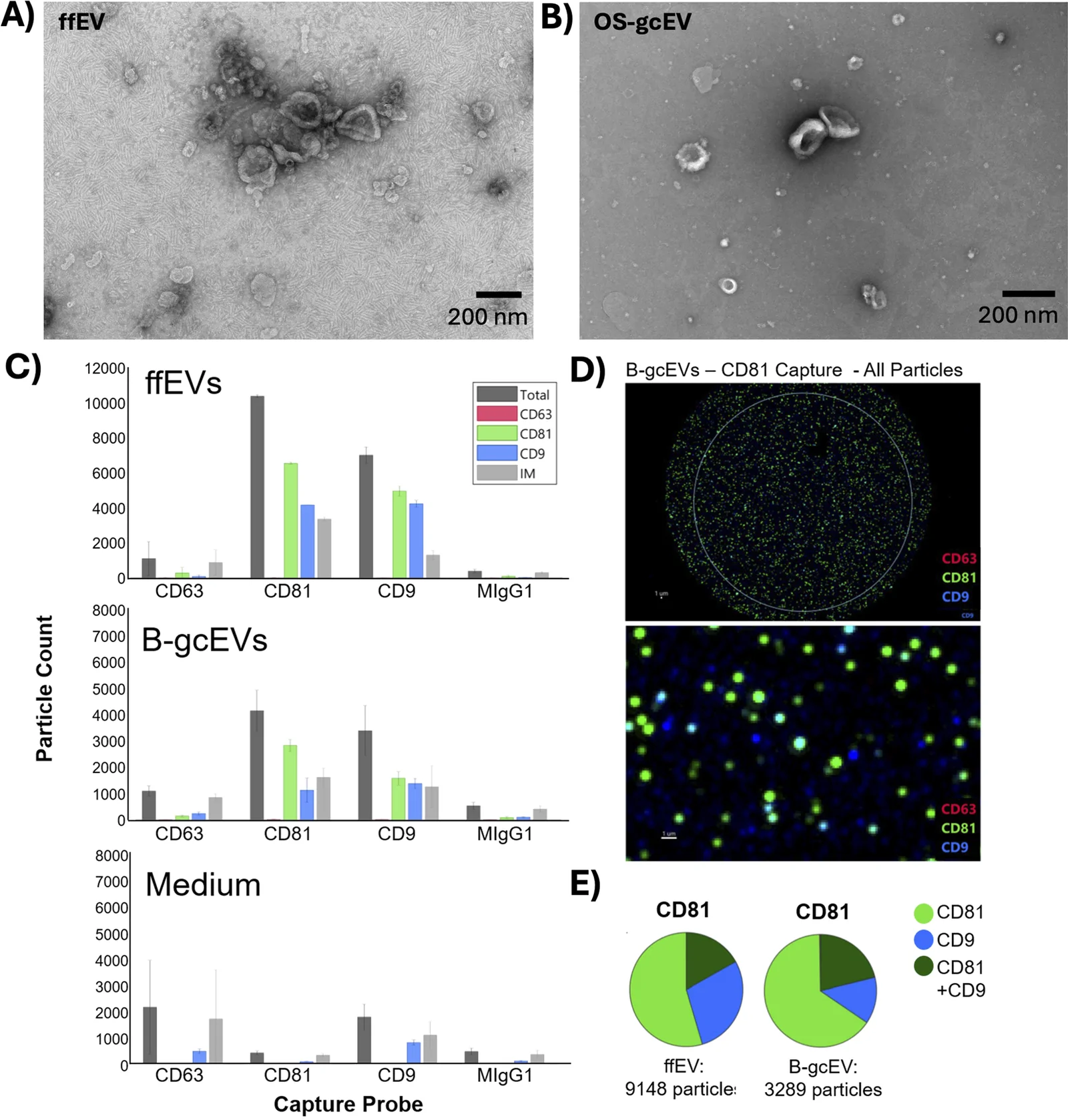
Transmission electron microscopy and EV tetraspanin characterization via Leprechaun Chip Array analysis of domestic cat gcEVs derived from ovarian granulosa cells exposed to microfluidic culture with gonadotropin stimulation and native ffEVs. **(A, B)** are representative TEM images for both ffEV and gcEVs at 25,000x; **(C)** CD63, CD81,and CD9-positive and interference microscopy (IM) particle counts, captured by CD63, CD81, CD9 or isotype control (MIgG1) for ffEVs, gcEVs from the Baseline treatment group (B_gcEVs), and culture medium controls; **(D)** Example image capture demonstrating CD81 and CD9 signal and co-localization, but no cross-reactivity with CD63 above background; and **(E)** Piecharts of CD81 captured particles from ffEV and B-gcEV groups which were positive for CD81, CD9, or both. FfEV TEM image from n = 3 pooled biological replicates and OS-gcEV TEM image from n = 5 pooled biological replicates. FfEV, B_gcEV, and medium control Leprechaun Chip Assay from n = 1 each.

Leprechaun Chip Assay demonstrated CD81- and CD9-positive ffEVs and gcEVs (B_gcEV treatment group), whereas culture medium controls treated to the same EV isolation processing as spent medium did not identify any particles with positive markers for any of the tetraspanins evaluated (below isotype control) (Figures 4C,D). Of the CD81-positive EVs, 16.8% of ffEVs and 21.0% of gcEVs were also positive for CD9 (Figure 4E).

### Exposure to chip-derived gcEVs or native ffEVs for 24h does not impact granulosa cell viability, but changes gene expression

To assess the functionality of gcEVs produced via the microfluidic device under the various gonadotropin stimulation protocols (C_gcEV, B_gcEV, NE_gcEV, and OS_gcEV), additional granulosa cells (n = 3 biological replicates from pools of 5–6 cats/replicate, 2,500 cells seeded/well) were co-incubated with generated gcEVs or native ffEVs compared with M_EV for 24 h. A scratch was made in each well to investigate the impact of EV presence on granulosa cell migration (Figure 5A). Additionally, 1250 cells were seeded/well and exposed to each corresponding gcEV treatment group for 48 h to assess possible gene expression changes. For the scratch assay cells, granulosa cell viability was consistent across treatment groups (Figure 5B), and the percentage of the total cell population per treatment and replicate in the wound area at 24 h did not differ between groups (Figure 5C). Though no statistical differences were observed, the NE_gcEV and OS_gcEV supplemented treatment groups had two of the highest values of cells within the wound area at 24 h, similar to ffEV supplemented cells. Additionally, the non-scratch assay incubated cells were collected for RNA isolation, reverse transcription, and qPCR analysis for *CYP19A1, CASP3, and STAR* expression to further assess EV functionality. No statistical differences were observed among treatment groups for *CYP19A1* expression and were highly variable, but significant differences were present between OS_gcEV and M_EV for *CASP3* (*p* = 0.0410) and both the ffEV and B_gcEV groups compared to M_EV for *STAR* (*p* = 0.0288 and 0.0034, respectively) (Figures 5D–F).

**FIGURE 5:**
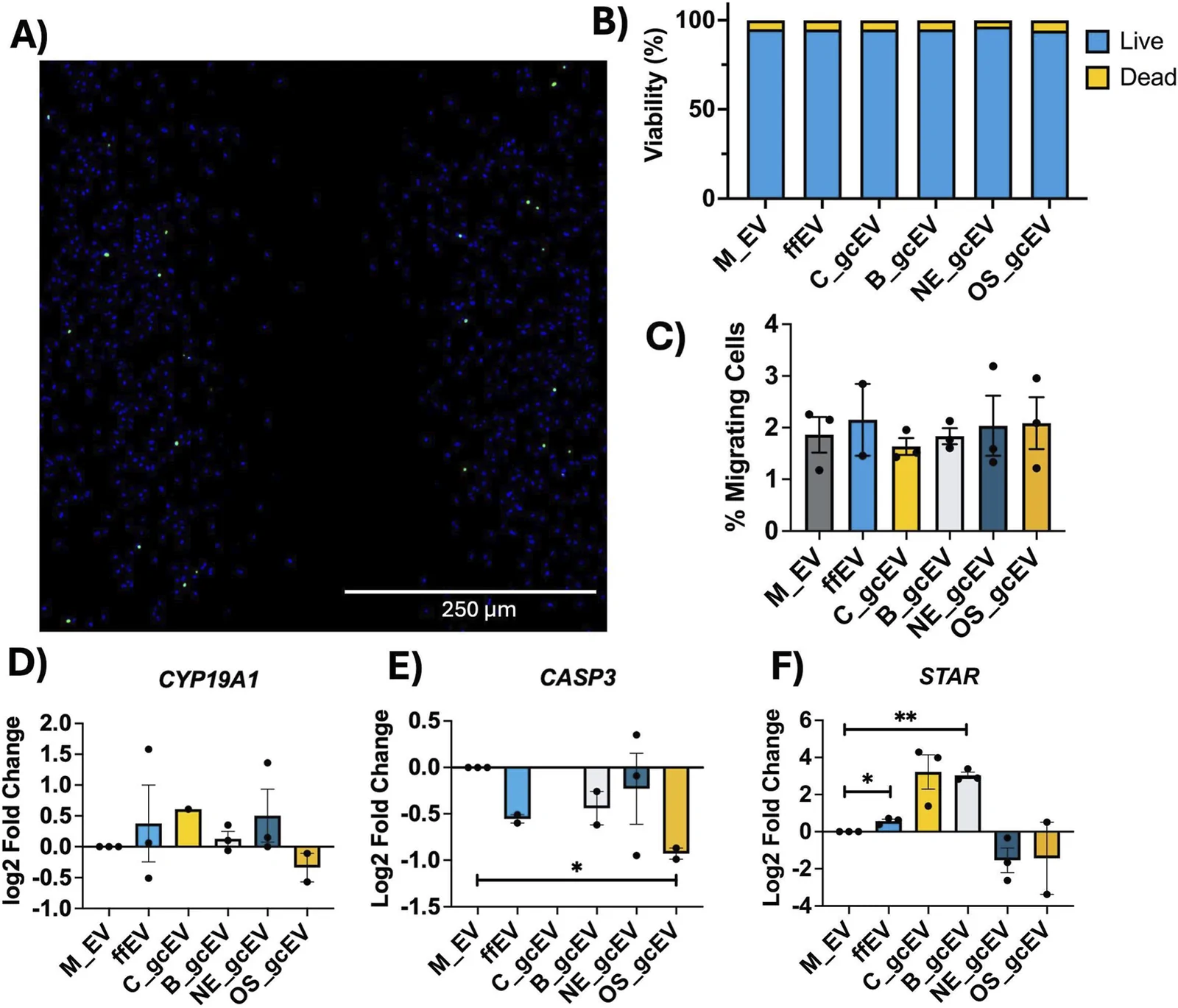
Bioactivity assessments of gcEVs derived from ovarian granulosa cells exposed to Control (C_gcEV), Baseline (B_gcEV), Natural Estrus-mimetic (NE_gcEV), and Ovulation induction-mimetic (OS_gcEV) gonadotropin protocols compared with corresponding ffEVs and medium only controls (M_EV), with **(A)** representative image of granulosa cell scratch assay viability at post-24-h incubation (representative image of C_gcEV treated granulosa cells, blue = live, green = dead), **(B)** recipient granulosa cell viability per treatment group post-scratch assay, **(C)** fraction of granulosa cells found within the wound area at 24 h after gcEV exposure, and **(D–F)** expression of *CYP19A1, CASP3,* and *STAR* in granulosa cells following exposure to gcEVs. Graphs are mean ± SEM and * = p < 0.05, ** = p < 0.01.

## Discussion

Granulosa cells play a critical role in ovarian function, supporting oocyte development and regulating the intrafollicular microenvironment through hormone production and EV signaling. While microfluidic systems have increasingly been used to model ovarian physiology, most work has focused on recreating whole follicles or multicellular structures (Wu et al., 2024; Pan et al., 2025; Malo et al., 2024; Healy et al., 2021; Yang et al., 2025; Won et al., 2019; Xiao et al., 2017; Del Valle et al., 2022; Barbato et al., 2023) with comparatively little emphasis on granulosa cell-specific platforms. Moreover, as interest in reproductive EVs for enhancing ARTs grows, there is increasing attention to systems which may support controlled production of functionally relevant EVs. In this study, we develop a microfluidic system to model biomimetic gonadotropin exposure and characterize granulosa cell responses and associated gcEVs. The system supports sustained and dynamic hormone delivery, enabling simulation of both natural estrus patterns and ovarian stimulation regimens *in vitro*. We demonstrate that granulosa cell viability is maintained within our microfluidic culture system environment, and that cells remain responsive to hormonal stimulation across treatment conditions. GcEVs exhibited particle size distribution, morphology, and tetraspanin biomarkers comparable to native ffEVs, despite lower overall concentrations. Furthermore, supplementation of gcEVs generated under different gonadotropin stimulation protocols to naive granulosa cells in culture altered gene expression profiles, even under short-term incubation. Together, these findings suggest this platform provides a controlled approach to investigate granulosa cell responses and EV cargo under dynamic hormonal conditions, offering a new opportunity to better understand how hormone-driven changes in the granulosa cell microenvironment may influence downstream oocyte competence.

Granulosa cell steroidogenic activity is regulated by progressive FSH and LH signals throughout follicular development, providing a rationale for modeling dynamic gonadotropin exposure within the microfluidic system. However, despite differences in gonadotropin exposure among treatment groups, there were no significant differences observed in granulosa cell progesterone production. Progesterone concentrations increased from 48 to 108 h across all gonadotropin treatment groups compared with 0–48 h, demonstrating the steroidogenic functionality of granulosa cells in the microfluidic chip. This included the control treatment group, which was not exposed to gonadotropins over the incubation, indicating that cat granulosa cells from antral follicles maintain basal steroidogenic function, even after two passages. Intriguingly, post-culture granulosa cells in the B, NE, and OS treatment groups had a more granular appearance compared to those in C, with NE and OS exhibiting the most prominent granular appearances. It is well known that LH increases lipid droplet accumulation in granulosa cells to shift cells towards prioritizing progesterone production, thus requiring high amounts of rapid-mobilization precursors for high-volume steroidogenesis to support early pregnancy (Talbott et al., 2020; Plewes et al., 2023; Plewes et al., 2020; Jensen et al., 2025). In our system, the peak LH surge or hCG stimulus occurred at 49 h of incubation, which if sufficiently bio-mimetic, should have triggered granulosa cell luteinization during the remaining culture period. Lipid droplet accumulation in the granular-appearing granulosa cells was confirmed with Oil Red O staining on a small subset of post-microfluidic culture cells and validated in oleic acid-supplemented, statically cultured cat granulosa cells (Supplementary File 4). While additional investigation is necessary to support this interpretation, the observed lack of differences in cell number and progesterone production in gonadotropin-stimulated groups represents an intriguing potential model to explore individual differences in granulosa cell response to gonadotropins and/or ovulation induction protocols in women for whom luteal insufficiency is a barrier to pregnancy success.

Unlike progesterone, 17-β-estradiol was not detected at any timepoint nor in any treatment group, as anticipated in the absence of theca cells (Hillier et al., 1994). While for the purposes of the present study this was positive evidence for the absence of theca cell contamination in our cultures, further evaluation of follicular cell functionality in our system would benefit from either co-culture or additional supplementation to assess full steroidogenic responsiveness. Steroidogenic activity is influenced by multiple factors, including granulosa cell differentiation state, culture conditions and duration, gonadotropin exposure, and availability of steroidogenic substrates (Fowler et al., 1978; Dorrington et al., 1983; Chiara et al., 2021). Thus, the absence of detectable estradiol in the present study cannot be attributed to gonadotropin concentration alone and suggests that additional optimization of the culture environment may be necessary to support a more complete steroidogenic phenotype. Recent work explored the addition of androstenedione to human granulosa-lutein cells to investigate the potential of extending the pre-luteinization period, and this concept would be intriguing in our culture system as well to identify potential rescue of estradiol production, given the potential luteinization characteristics we observed (Jensen et al., 2025). Additional future work includes incorporating a longitudinal assessment of estradiol and progesterone production alongside steroidogenic gene expression, which will help determine how granulosa cell steroidogenic activity develops over microfluidic culture and in response to dynamic gonadotropin profiles.

While the benefits of microfluidic cell culture are recognized, it is similarly known that resulting shear stress on cells can alter their physiology and function (Hyler et al., 2018). Cells in our system were exposed to very low levels of shear stress <0.01 dyne/cm^2^ (as compared with other mechano-sensitive cells such as kidney proximal tubule cells with 0.2 dyne/cm) (Jang et al., 2013). However, we did not observe adverse effects of fluid flow/shear stress on granulosa cells incubated in the microfluidic device, with cell viability above 88% in all groups at the end of microfluidic culture and no evidence of increased apoptotic bodies (per NTA) of gcEVs in our culture system compared with ffEVs. Additionally, it is known that stressed (heat or oxidative) bovine granulosa cells produce EVs that deliver bioactive molecules to recipient cells to promote survival or transfer pro-apoptotic messages/damage signals that lead to activation of enzymes such as *CASP3* (Menjivar et al., 2023; Gebremedhn et al., 2020; Khamari et al., 2026; Diaz-Muñoz et al., 2026; Abramowicz et al., 2019). As granulosa cell survival after gcEV exposure was an important factor to consider, we tested *CASP3* expression and observed a general reduction in our EV-treated granulosa cells. This was not significant and was equivalent to the direction of change in ffEVs, so unlikely to be the result of shear stress influence. However, the significant decrease in *CASP3* in the OS_gcEV treated cells relative to M_EV joins earlier evidence of the influence of gonadotropins over EV molecular cargo that requires deeper investigation (Drula et al., 2023; Sandoval, 2025; Kargl et al., 2026; Bi et al., 2026).

An unexpected observation in the present study was the difficulty in detaching granulosa cells from the microfluidic device following culture under flow conditions. In initial static cultures, traditional 0.5% trypsin + EDTA (3–5 min at 37 °C) was highly efficient at detaching cells and maintaining their viability, yet in the microfluidic chip following incubation, the granulosa cells would lyse after 2 min of trypsin incubation releasing DNA contents. Anecdotally, cells were also more likely to detach as a sheet, even when not at full confluence in the chip. Although direct comparisons of granulosa cell gap junction behavior between static and microfluidic culture platforms are limited, accumulating evidence indicates that granulosa cell connectivity is highly sensitive to the physical properties of the culture environment. For example, three-dimensional granulosa cell microtissues have been shown to develop multilayered architectures with robust connexin-43 expression and active gap junction intercellular communication, more closely resembling native follicular organization (Ip et al., 2022). This highlights the inherent plasticity of granulosa cell networks and demonstrates that deviations from standard static conditions may promote the formation of more physiologically relevant intercellular coupling. In parallel, recent studies have shown that both follicular fluid components and hormonal stimulation can modulate connexin-43 expression and gap junction communication *in vitro* (Marin et al., 2025; Zhang et al., 2026). In other cell types, microfluidic culture systems have been shown to enhance intercellular interactions and signaling dynamics, as well as provide controlled methods to study these precise interactions (Pan et al., 2025; Pang et al., 2021). Though gap junction differences between static and microfluidic cultured cells were not explicitly demonstrated in this study, we postulate that the increased adhesion and resistance to detachment observed under flow conditions may reflect enhanced intercellular coupling and the establishment of more integrated granulosa cell networks. While additional investigation is needed, such changes could have downstream implications for coordinated signaling, including both gap junction-mediated communication and EV exchange within follicular microenvironments.

Toward the goal of producing high concentrations of functionally relevant EVs from our microfluidic culture system, the functional capacity of generated gcEVs on naive granulosa cells was investigated. As noted earlier, it was encouraging that granulosa cells cultured in the presence of the EVs maintained high viability and demonstrated similar migration rates as controls. Hormone production, specifically estradiol and later progesterone output, is an important function of granulosa cells, thus why we investigated *CYP19A1* and *STAR* expression in granulosa cells after gcEV co-incubation. *CYP19A1* expression exhibited only modest and variable changes across treatments, which was in alignment with the lack of detectable estradiol production by the gcEV-generating cells in the microfluidic chip. Conversely, gcEVs produced under different hormonal environments elicited differing recipient cell *STAR* expression patterns, with reduced expression in NE_gcEV and OS_gcEV exposed cells compared with M_EV, though not statistically significant. The ffEV, C_gcEV, and B_gcEV exposed cells exhibited increased expression, with ffEV and B_gcEV reaching significance. This difference in expression pattern among the gonadotropin treatment groups leads to the hypothesis that a potential effect of luteinization is occurring. Granulosa cells can spontaneously luteinize in serum-supplemented culture conditions, and granulosa cells specifically collected from human small antral follicles express increasing levels of *STAR* and decreasing *CYP19A1* across 48 h of FSH-free culture (Jensen et al., 2025). Additionally, *STAR* expression is regulated under gonadotropin control, sharply rising after PMSG or hCG stimulation (San et al., 1996). These trends bring up future questions to address regarding how the LH-surge and luteinization change the molecular signature dynamics of EV cargo across our gonadotropin treatment groups, and furthermore, how that is translated to neighboring cells, such as COC uptake of gcEVs, which is a remaining gap in the field.

As an initial characterization of this microfluidic system, several factors should be considered when interpreting the present findings and optimizing future applications of the model. Although feline granulosa cells and intact antral follicles have previously demonstrated steroidogenic responses to gonadotropin stimulation within 12–48 h under static culture conditions (Chiara et al., 2021; Rojo et al., 2015), the optimal duration of dynamic gonadotropin exposures under microfluidic conditions remains unknown. The 4.5 days culture period used in the present study therefore represents an initial exposure window, and extending the culture duration in future iterations of the model may help determine the optimal culture duration to recapitulate more *in vivo* phenotypic and steroidogenic responses. Feline granulosa cells have been maintained in a 3D spheroid system for up to 11 days to study luteinization, so longer culture is possible (Hryciuk et al., 2023). The 4.5 days culture duration period was selected largely due to our preliminary study (Supplementary File 1) investigating follicle size on EV function. We demonstrated that when follicular fluid from pooled follicles >1 mm were co-incubated with immature domestic cat COCs, significant increases in cleaved embryos per MII oocyte were observed compared with unsupplemented controls. This led to targeting ≥1.5 mm follicles to collect granulosa cells, which have already been exposed to initial gonadotropin stimuli for subsequent incubation in the defined IVF-like gonadotropin environment during the late follicular/periovulatory period. Cell viability after 4.5 days of culture in the microfluidic device was high (>88%), but cell number did not differ among gonadotropin treatment groups, thus extending culture duration is plausible and may allow more pronounced phenotypic and steroidogenic changes to emerge.

Additionally, gonadotropin concentrations of the feline intrafollicular environment are not well characterized and human values were used as a starting framework in this study. More specifically, recombinant human gonadotropins have previously elicited steroidogenic and cumulus responses in cultured domestic cat antral follicles (Rojo et al., 2015). However, species-specific differences in gonadotropin exposure remain an important consideration, particularly given that LH secretion in the domestic cat varies according to the stage of estrus at the time of copulation and with the number of copulations (Santos and Silva, 2023). Both concentration and temporal pattern of LH exposure contribute to ovulation in the domestic cat (Chakraborty et al., 1979), which supports the use of microfluidic culture to investigate the dynamic exposure needed for more *in vivo* modeling.

Finally, like other efforts to generate EVs at scale across other disciplines (Cook and Li, 2025; Gudbergsson et al., 2016), there are concerns that 1) some minor contribution of residual EVs from the EV-depleted serum supplement may contaminate the EVs, and 2) generation of large quantities of EVs *in vitro* is needed for all downstream analyses. We were encouraged that little to no detectable EVs were observed via TEM in our EV-depleted serum supplement (Supplementary File 5), and that tetraspanin-positive particles were similarly below the detection threshold of the interference microscopy control in our media sample. Moreover, although NTA did detect particles in the size range of EVs, these exhibited lower X10, X50, X90, mean, and mode values than those observed for gcEVs and ffEVs. Toward the second point, while our gcEV collection was initiated in cells at 80% confluence, we also found gcEV production to be a limitation. It is well known that follicular fluid contains abundant EVs, with EV-enriched preparations from several mammalian species yielding particle concentrations on the order of 10^11^–10^12^ particles/mL by NTA (Navakanitworakul et al., 2016; Gabryś et al., 2024; Battaglia et al., 2020). As gcEVs represent only a portion of the ffEV population (Uzbekova et al., 2020), the same concentration is not expected between the two, yet gcEV concentration in our model requires enhancement for downstream experimental use. Future work will heavily focus on fabrication of a larger microfluidic device design to significantly increase the number of cells plated per chip, with the goal of generating higher gcEV concentrations as needed to evaluate gcEV functionality for clinical applications.

In conclusion, this study demonstrates that granulosa cells from domestic cat antral follicles (≥1.5 mm) can be isolated, remain viable in a microfluidic device for 4.5 days, produce progesterone, and generate more *in vivo*-like EVs that are capable of eliciting downstream recipient cell gene responses. The work presented here establishes a bio-mimetic gonadotropin stimulation and microfluidic culture to continue dissecting granulosa cell-specific responses to different hormonal conditions. This allows for novel insights into the molecular mechanisms regulating intrafollicular communication and oocyte maturation.

## Data Availability

The original contributions presented in the study are included in the article/Supplementary Material, further inquiries can be directed to the corresponding author.
